# Sex-Based Differences in Long-Term Outcomes Following Intravascular Brachytherapy for In-Stent Restenosis

**DOI:** 10.1016/j.jscai.2025.104041

**Published:** 2025-11-25

**Authors:** Mangesh Kritya, Chloe Kharsa, Gal Sella, Devin Olek, Bin S. Teh, Muhammad Faraz Anwaar, Joseph Elias, Elia El Hajj, Albert E. Raizner, Andrew Farach, Neal S. Kleiman, Alpesh R. Shah

**Affiliations:** a Department of Cardiology, Houston Methodist DeBakey Heart & Vascular Center, Houston, Texas; b Department of Radiation Oncology, Houston Methodist Hospital, Houston, Texas

**Keywords:** in-stent restenosis, intravascular brachytherapy, percutaneous coronary intervention, sex-based differences

## Abstract

**Background:**

In-stent restenosis (ISR) remains a challenging complication following percutaneous coronary intervention, and intravascular brachytherapy (IVBT) has proved to be an important treatment strategy. However, limited data exist on sex-specific outcomes following IVBT.

**Methods:**

This retrospective, single-center cohort study included 223 patients (61 women, 162 men) treated with IVBT for ISR between 2014 and 2023. The primary end points were all-cause mortality, target lesion revascularization, and major adverse cardiovascular events. Secondary outcomes included technical success, myocardial infarction, cardiac death, and heart failure hospitalization. Multivariable Cox regression was used to adjust for clinical and procedural covariates.

**Results:**

Baseline characteristics were largely similar between sexes, except for higher body surface area and diabetes prevalence in men. Procedural success rates did not differ between groups. However, female sex was independently associated with a higher risk of target lesion revascularization (adjusted HR, 1.80; 95% CI, 1.07-3.01; *P* = .026) and major adverse cardiovascular events (adjusted HR, 1.63; 95% CI, 1.09-2.45; *P* = .017). Women also had a higher risk of myocardial infarction (adjusted HR, 2.58; 95% CI, 1.29-5.19; *P* = .008), whereas no significant sex-based differences were observed for all-cause mortality or heart failure hospitalization.

**Conclusions:**

Despite comparable procedural outcomes, women undergoing IVBT for ISR experienced higher rates of adverse cardiovascular events. These findings underscore the need for sex-stratified risk assessment and further prospective research to understand and address sex-based differences in outcomes after IVBT.

## Introduction

In-stent restenosis (ISR) remains a problem following percutaneous coronary intervention (PCI) with drug-eluting stents (DES). ISR is characterized by the renarrowing of the stented segment, typically due to neointimal hyperplasia, and can lead to clinical manifestations such as recurrent angina or acute coronary syndromes, often necessitating repeat revascularization procedures. The prevalence of ISR in the era of DES ranges from 5% to 10% among patients undergoing PCI, although this can vary based on patient and lesion characteristics.^1^, ^2^, ^3^

Intravascular brachytherapy (IVBT) involves the delivery of localized radiation to the stented segment to inhibit neointimal proliferation and reduce the likelihood of restenosis. It was initially utilized for the treatment of bare-metal stent restenosis^4^^,^^5^ with the advent and widespread adoption of DES, its use declined, only to be revisited recently as an important strategy for managing DES-ISR. Current ACC/AHA guidelines recommend IVBT as a viable treatment option for ISR,^1^ supported by evidence showing its effectiveness in reducing rates of target lesion revascularization (TLR) and major adverse cardiovascular events (MACE).^5^, ^6^, ^7^, ^8^

Women remain underrepresented in clinical cardiovascular trials,^9^^,^^10^ and this limits our understanding of sex-specific differences in response to revascularization strategies.^11^ For IVBT, available evidence suggests that women may experience different outcomes after revascularization procedures compared to men.^12^^,^^13^ Despite the demonstrated efficacy of IVBT, no large-scale registry studies have systematically compared outcomes after IVBT based on sex. Addressing this gap may help form more specified revascularization strategies and improve clinical outcomes for both men and women.

This study aims to compare procedural and long-term outcomes between men and women undergoing IVBT for ISR using data from the Houston Methodist brachytherapy registry.

## Materials and methods

### Study design and population

This was a retrospective, single-center cohort study evaluating procedural and long-term clinical outcomes following IVBT for ISR. Patients were eligible for inclusion if they were aged ≥18 years and underwent IVBT for ISR in a native coronary artery or bypass graft with a reference vessel diameter between 2.7 mm and 4.0 mm. Exclusion criteria included multivessel coronary disease requiring PCI in multiple vessels or a history of prior chest radiation therapy. Of the 227 patients initially identified, 223 met the inclusion criteria and comprised the final cohort. Baseline characteristics and clinical outcomes were extracted from electronic medical records.

In-stent restenosis was defined as angiographically documented luminal narrowing ≥50% within a previously stented segment, accompanied by objective evidence of myocardial ischemia. Patients were stratified by sex for comparison of clinical outcomes. Abnormal congestive heart failure (CHF) has been defined as EF <50%. Technical success was defined as the accurate deployment of the radiation delivery system at the target lesion and the complete delivery of the prescribed dose without any immediate procedural complications. Patients treated for acute MI at the index presentation were not candidates for IVBT during that admission and were therefore not included.

### Study end points

The primary end points included all-cause mortality, TLR, and MACE, defined as a composite of cardiac death, target vessel myocardial infarction (MI), thrombosis, bleeding, and chest pain. Secondary end points included technical success rates, individual adverse events, MI, cardiac death, and hospitalization due to heart failure. TLR was defined as any repeat percutaneous intervention or surgical bypass of the target lesion performed for restenosis within 1 year of the index procedure.

### Brachytherapy procedure

All patients underwent diagnostic angiography before IVBT. Intravascular ultrasound (Eagle Eye, Philips Volcano) was used at the discretion of the operator for lesion assessment and planning. Before brachytherapy, patients underwent repeat PCI. The procedures were performed according to the vendor-recommended protocol and, when intravascular ultrasound was utilized, neointimal proliferation was recorded as a suspected ISR mechanism.

In complex ISR lesions, adjunctive plaque modification techniques including laser atherectomy, shockwave intravascular lithotripsy, cutting, and scoring balloons were employed at the operator's discretion to optimize lesion preparation.

Procedures were performed according to the vendor-recommended protocol. After successful balloon preparation of the restenotic lesion, beta radiation was delivered using a 7F guide catheter with the Beta-Cath 3.5F System (Novoste Corporation). The source train is comprised of strontium/yttrium-90 seeds. To ensure complete lesion coverage and account for edge effects, the radiation source train length was selected to exceed the angioplasty segment by 10 mm on each end. Due to guide catheter placement issues or specific patient conditions, reduced margins were accepted in selected cases. Radiation dosing followed a vessel size-dependent protocol, in order to deliver a uniform dose to the tissue in all cases, with all doses prescribed at 2 mm from the radioactive source center: vessels with diameters ≤3.35 mm received 18.4 Gy, whereas vessel with diameters >3.35 mm received 23 Gy. Long lesions that required multiple contiguous dwells had an overlap segment of approximately 10 mm.

Periprocedural anticoagulation was with IV unfractionated heparin (70 IU/kg or 5000 IU) or bivalirudin (0.75 mg/kg bolus then 1.75 mg/kg/h). At Houston Methodist, bivalirudin was preferred. Supplemental dosing was given as needed to maintain therapeutic anticoagulation. Postprocedure, patients received aspirin 75 to 100 mg daily indefinitely and a P2Y12 inhibitor, typically clopidogrel 75 mg/d after a 300 to 600 mg load for ≥12 months; prasugrel or ticagrelor could be substituted per contemporaneous guidelines and clinical judgment.^1^^,^^14^, ^15^, ^16^

### Statistical analysis

Baseline characteristics were summarized using means and standard deviations for continuous variables, and comparisons between sexes were performed using *t* test. Categorical variables were presented as count (%) and compared using χ^2^ tests or Fisher exact test when expected frequencies were small. For clinical outcomes, time-to-event analysis was performed and visualized using Kaplan-Meier survival curves, with differences assessed using the log-rank test. Univariate Cox proportional hazards analysis was conducted to estimate hazard ratios (HR) with corresponding 95% CI; a 2-tailed *P* value < .05 was considered statistically significant.

Multivariable Cox proportional hazards regression was used to adjust for potential confounders, reporting HR with 95% CI. Two models were constructed for comparison. The first model adjusted for clinical comorbidities, including age, diabetes mellitus, chronic kidney disease, hypertension, hyperlipidemia, chronic obstructive pulmonary disease, and CHF. The second model incorporated available procedural variables, including preprocedural left ventricular ejection fraction, balloon diameter, balloon length, vessel diameter, and lesion length. A 2-tailed *P* value <.05 was considered statistically significant. All statistical analyses were performed using R version 4.4.2 (R Foundation for Statistical Computing).

Target lesion revascularization was prespecified as the primary end point and tested at a 2-sided α = 0.05. Secondary end points (MACE, all-cause mortality, MI, heart failure hospitalization [HFH], and cardiac death) were analyzed with control of the false discovery rate (FDR) using the Benjamini-Hochberg procedure (q = 0.05); FDR-adjusted *P* values are reported within each model.

This manuscript was prepared in accordance with the Sex and Gender Equity in Research (SAGER) guidelines^17^ to ensure appropriate consideration of sex-based analyses.

## Results

A total of 223 patients were included, comprising 61 women and 162 men. The mean age was comparable between sexes (64.2 ± 12.4 years in women vs 65.8 ± 10.0 years in men; *P* = .405). Men had greater body surface area (2.08 ± 0.29 m^2^ vs 1.84 ± 0.26 m^2^; *P* < .001). The prevalence of hypertension (96.9% in men vs 95.1% in women; *P* = .512) and hyperlipidemia (93.2% vs 88.5%; *P* = .252) was similar across groups. Diabetes was more prevalent among men (68.5% vs 54.1%; *P* = .045). The baseline characteristics are provided in Table 1.

**Table 1:** Baseline characteristics.

Characteristics	Women (n = 61)	Men (n = 162)	*P* value
Age, y	64.2 ± 12.4	65.8 ± 10.0	.405
Body mass index, kg/m^2^	29.3 ± 12.4	30.7 ± 7.21	.30
Body surface area, m^2^	1.84 ± 0.26	2.08 ± 0.29	<.001
Hypertension	58 (95.1%)	157 (96.9%)	.512
Hyperlipidemia	54 (88.5%)	151 (93.2%)	.252
Diabetes	33 (54.1%)	111 (68.5%)	.045
Smoking	17 (27.9%)	54 (33.3%)	.435
Chronic kidney disease	18 (29.5%)	36 (22.2%)	.258
Dialysis	5 (8.2%)	10 (6.2%)	.591
COPD	4 (6.6%)	15 (9.3%)	.519
Abnormal EF	13 (21.3%)	42 (25.9%)	.476

Values are mean ± SD or n (%).

COPD, chronic obstructive pulmonary disease; EF, ejection fraction.

### Procedural characteristics

Procedural features were largely comparable between women and men, as detailed in Table 2. The left anterior descending artery was the most commonly treated vessel in each group (36.5% in women vs 30.1% in men; *P* = .439), with no sex-based differences in the distribution of other target vessels. Vessel diameter (3.44 ± 0.46 mm vs 3.46 ± 0.46 mm; *P* = .770) and injury length (25.72 ± 16.75 mm vs 28.93 ± 18.86 mm; *P* = .215) were also similar between sexes. The frequency of calcified lesions (4.8% vs 4.9%; *P* > .99) and tortuous anatomy (1.6% vs 1.8%; *P* > .99) was low and not significantly different.

**Table 2:** Procedural characteristics.

Characteristics	Women (n = 61)	Men (n = 162)	*P* value
Unstable angina	36 (57.1%)	70 (42.9%)	.076
Target vessel			
Left anterior descending artery	23 (36.5%)	49 (30.1%)	.439
Left circumflex artery	14 (22.2%)	44 (27.0%)	.571
Right coronary artery	17 (27.0%)	45 (27.6%)	>.99
Ramus intermedius	1 (1.6%)	5 (3.1%)	>.99
Saphenous vein graft	3 (4.7%)	3 (1.8%)	.352
Left main artery	6 (9.5%)	8 (4.9%)	.325
Right internal mammary artery	0 (0%)	1 (0.6%)	>.99
Vessel diameter, mm	3.44 ± 0.46	3.46 ± 0.46	.770
Lesion length, mm	20.87 ± 16.65	27.78 ± 18.86	<.001
Injury length, mm	25.72 ± 16.75	28.93 ± 18.65	.215
Calcified lesion	3 (4.8%)	8 (4.9%)	>.99
Tortuosity	1 (1.6%)	3 (1.8%)	>.99
Lesion preparation			
Noncompliant balloon	26 (41.3%)	94 (57.7%)	.038
Semicompliant balloon	8 (12.7%)	9 (5.5%)	.120
Scoring balloon	6 (9.5%)	3 (1.8%)	.016
Cutting balloon	22 (34.9%)	48 (29.4%)	.524
Laser/intravascular lithotripsy	1 (1.6%)	6 (3.7%)	.676
Balloon diameter used for preparation, mm	3.49 ± 0.65	3.55 ± 0.60	.526
Balloon length used for preparation, mm	13.93 ± 3.76	13.78 ± 3.54	.785
Contrast volume, mL	155 ± 59.73	145 ± 57.82	.257
Procedure length, min	91.56 ± 26.62	91.95 ± 43.07	.935
Technical success	63 (100%)	162 (99.4%)	.91

Values are mean ± SD or n (%).

Some variation was observed in lesion preparation techniques. Noncompliant balloons were used more frequently in men (57.7% vs 41.3%; *P* = .038), whereas scoring balloons were more often used in women (9.5% vs 1.8%; *P* = .016). The use of semicompliant balloons, cutting balloons, and plaque modification devices such as laser or shockwave therapy was similar across groups. There were no significant differences in balloon diameter or length used for lesion preparation. Lesion length was significantly longer in men as compared to women (27.78 ± 18.86 mm vs 20.87 ± 16.65 mm; *P* < .001). Contrast volume and overall procedure time were also comparable between sexes. Technical success was achieved in all women and in 99.4% of men (*P* = .91), with no significant difference observed.

### TLR

**Figure 1:**
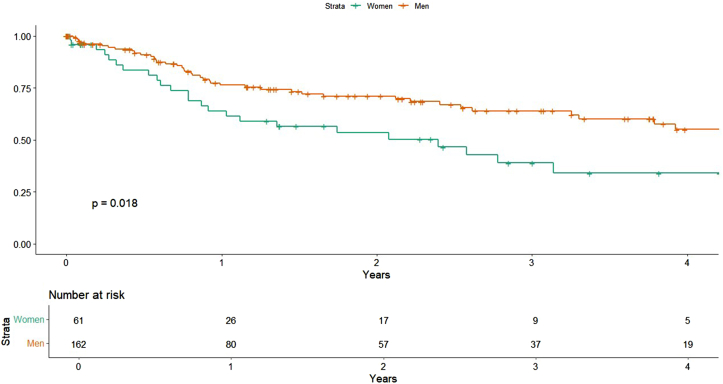
**Target lesion revascularization by sex.**

In univariable analysis, women exhibited a significantly higher risk of TLR compared with men (HR, 1.81; 95% CI, 1.10-3.00; *P* = .018) (Figure 1). This association persisted after adjusting for comorbidities (HR, 1.97; 95% CI, 1.18-3.29; *P* = .0095) and procedural variables (HR, 1.92; 95% CI, 1.14-3.24; *P* = .014), indicating an independent association between female sex and increased TLR risk.

### MACE

**Figure 2:**
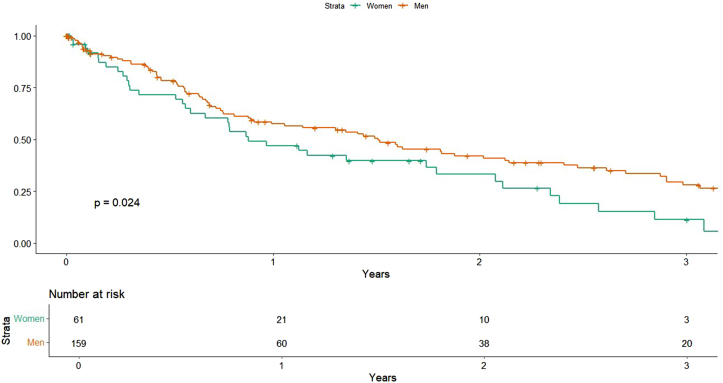
**Major adverse cardiovascular events by sex.**

Women had a significantly greater risk of MACE in univariable analysis (HR, 1.57; 95% CI, 1.06-2.33, *P* = .024) (Figure 2). This finding remained significant in both comorbidity-adjusted (HR, 1.56; 95% CI, 1.04-2.35; *P* = .033) and procedural-adjusted models (HR, 1.73; 95% CI, 1.15-2.61, *P* = .008), suggesting a consistent sex-based difference in long-term composite cardiovascular outcomes.

### All-cause and cardiac mortality

**Figure 3:**
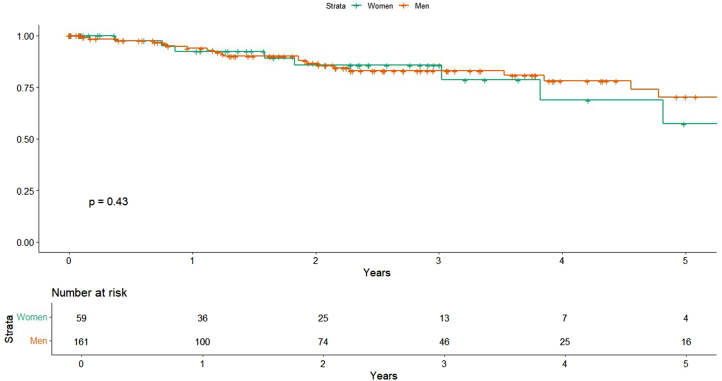
**All-cause mortality by sex.**

**Figure 4:**
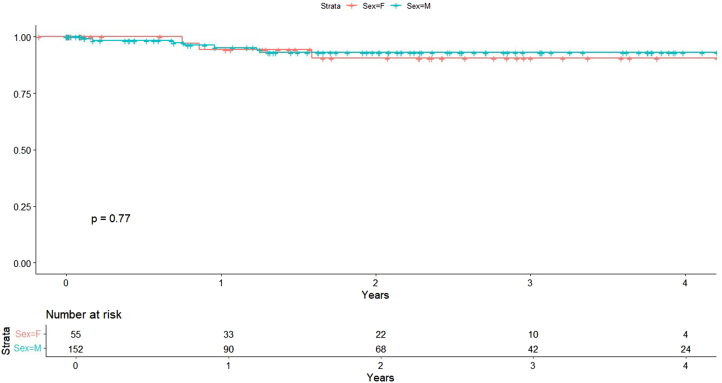
**Cardiac death by sex.**

There was no difference in all-cause mortality between groups in the univariate model (HR, 1.35; 95% CI, 0.64-2.87; *P* = .428) (Figure 3), nor after adjustment for comorbidities (HR, 1.57; 95% CI, 0.71-3.48; *P* = .262) or procedural variables (HR, 1.60; 95% CI, 0.73-3.51; *P* = .237). Among comorbidities, chronic obstructive pulmonary disease was significantly associated with increased mortality risk (HR, 3.05; 95% CI, 1.21-7.64, *P* = .018) (Table 3). Cardiac-specific mortality was similarly not associated with sex (HR, 1.22; 95% CI, 0.21-3.17; *P* = .773) in univariate analysis (Figure 4).

**Table 3:** Independent predictors after multivariable adjustment.

Predictor	Outcome	HR (95% CI)	*P* value
COPD	All-cause mortality	3.05 (1.21-7.64)	.018
CHF	HFH	25.10 (3.03-207.40)	.0028
LVEF (%)	HFH	0.95 (0.91-0.98)	.003
CHF	MI	2.38 (1.07-5.29)	.034

CHF, congestive heart failure; COPD, chronic obstructive pulmonary disease; HFH, heart failure hospitalization; HR, hazard ratio; LVEF, left ventricular ejection fraction.

### MI

**Figure 5:**
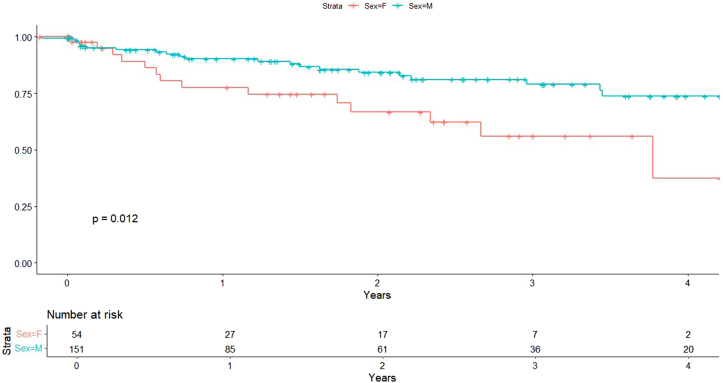
**Myocardial infarction by sex.**

The incidence of MI was higher in women across all models. Univariable analysis demonstrated an elevated risk (HR, 2.34; 95% CI 1.18-4.63; *P* = .012) (Figure 5), which persisted after adjustment for comorbidities (HR, 2.18; 95% CI, 1.02-4.65; *P* = .044) and procedural characteristics (HR, 2.66; 95% CI, 1.32-5.58; *P* = .006). CHF (HR, 2.38, *P* = .034) was also independently associated with MI risk in multivariate models.

### HFH

**Figure 6:**
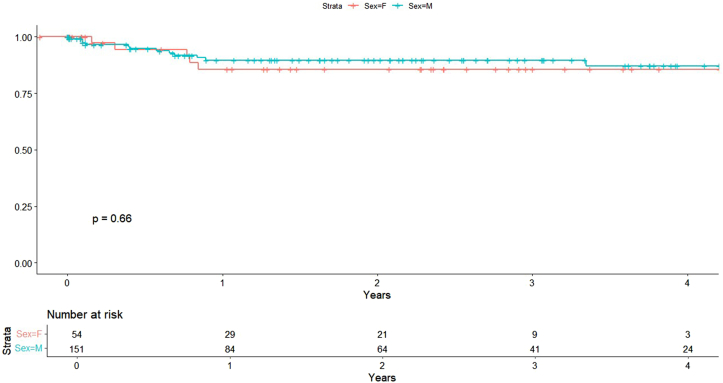
**Heart failure hospitalizations by sex.**

There was no significant association between sex and the risk of HFH in either univariate (HR, 1.26; 95% CI, 0.44-3.59; *P* = .662) (Figure 6) or multivariate analyses adjusted for comorbidities (HR, 1.30; 95% CI, 0.40-4.20; *P* = .664) and procedural variables (HR, 1.34; 95% CI, 0.44-4.10; *P* = .600).

After controlling the FDR across secondary end points, associations for MACE and MI remained significant only in the procedural variables model (both FDR-adjusted *P* = .016), whereas other secondary end points were not significant after adjustment (Table 4). Among all covariates, reduced echocardiographic left ventricular ejection fraction was independently associated with HFH in the procedural model (HR, 0.95 per % increase; 95% CI, 0.91-0.98; *P* = .003). CHF was the strongest predictor in the comorbidity-adjusted model (HR, 25.1; 95% CI, 3.03-207.4; *P* = .0028) (Table 3). The important findings of this paper have been summarized in the Central Illustration.

**Table 4:** Summary of all outcomes.

Outcome	Adjustment	HR (95% CI)	*P* value
TLR	Unadjusted	1.81 (1.10-3.00)	.020
	Comorbidities	1.97 (1.18-3.29)	.001
	Procedural variables	1.92 (1.14-3.24)	.014
MACE	Unadjusted	1.57 (1.06-2.33)	.026
	Comorbidities	1.56 (1.04-2.35)	.033
	Procedural variables	1.74 (1.15-2.61)	.008^a^
All-cause mortality	Unadjusted	1.35 (0.64-2.87)	.428
	Comorbidities	1.57 (0.71-3.48)	.262
	Procedural variables	1.60 (0.73-3.51)	.237
MI	Unadjusted	2.34 (1.18-4.63)	.014
	Comorbidities	2.18 (1.02-4.65)	.044
	Procedural variables	2.67 (1.32-5.38)	.006^a^
HFH	Unadjusted	1.26 (0.44-3.59)	.662
	Comorbidities	1.30 (0.40-4.20)	.664
	Procedural variables	1.34 (0.44-4.10)	.600
Cardiac mortality	Unadjusted	1.22 (0.21-3.17)	.773

Adjusted variables: Comorbidities: age, hypertension, hyperlipidemia, chronic kidney disease, congestive heart failure, chronic obstructive pulmonary disease, diabetes mellitus. Procedural variables: Preprocedural LVEF, balloon diameter, balloon length, vessel diameter, lesion length.

Primary end point: TLR tested at α = 0.05 without multiplicity adjustment. Secondary end points: *P* values adjusted using Benjamini-Hochberg false discovery rate (q = 0.05) within each model.

HFH, heart failure hospitalization; HR, hazard ratio; MACE, major adverse cardiovascular events; MI, myocardial infarction; TLR, target lesion revascularization.

a FDR–adjusted *P* < .05.

**Central Illustration:**
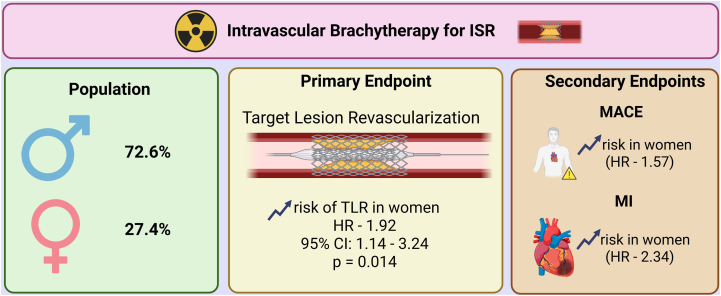
**Summary of sex-based outcomes after intracoronary brachytherapy for in-stent restenosis (ISR).** HR, hazard ratio; MACE, major adverse cardiovascular event; MI, myocardial infarction; TLR, target lesion revascularization.

## Discussion

In this study that evaluated sex-based differences in outcomes following IVBT for ISR, women demonstrated significantly higher risks of TLR, MI, and MACE compared to men. After adjusting multiplicity in secondary end points with FDR, MACE and MI remained significant only in the procedural variables model (both FDR-adjusted *P* = .016), and effects in other models were directionally similar but not significant after adjustment. In contrast, no statistically significant differences were observed between sexes for all-cause mortality, cardiac mortality, or HFH.

These findings align with previously observed patterns in which women experience higher rates of adverse cardiovascular events. Although sex-specific outcomes following IVBT remain underreported, a pooled analysis with multivariate adjustment of 21 randomized controlled trials (RCT) has shown that women had higher rates of MI and TLR at 5 years of follow-up post-PCI, which highlights worse outcomes of the female population.^18^ Although studies such as that by Cheng et al^19^ have shown increased MACE in IVBT-treated patients, sex-specific data remain lacking. Similarly, although women experience higher HFH rates following acute coronary events,^20^ corresponding data in the IVBT population are sparse. The overall frequency of MI in this cohort was lower than that in some other studies evaluating brachytherapy outcomes, possibly due to the longer duration of follow-up in their study.^21^

The observed sex-based differences in outcomes following IVBT, particularly the increased risk of TLR and MACE among women, are likely attributable to underlying pathophysiological mechanisms rather than procedural factors. In this study, baseline and procedural characteristics were generally comparable between sexes, including lesion location, injury length, balloon size, and diameter, as well as vessel diameter, despite the trend of smaller vessel size in women in the general population.^18^^,^^22^^,^^23^ However, the lesion length was significantly longer in men as compared to women. Along with the nearly equal technical success rate across groups, none of the evaluated procedural characteristics independently predicted any of the outcomes under investigation, ruling out procedural complexity as a possible explanation for outcome variations.

The worst outcomes observed in women may possibly be explained by multiple factors, sex-specific vascular biology being an important one. With a mean age of 64 years in this cohort, the majority of female patients were postmenopausal and subject to the effects of declining estrogen levels.^24^^,^^25^ The reduction in estrogen impairs endothelial function and diminishes vascular healing capacity, which may predispose women to restenosis following intervention.^26^^,^^27^ Estrogen also regulates vascular remodeling through estrogen receptor α and G-protein–coupled estrogen receptor, both of which influence smooth muscle proliferation and endothelial cell migration; the disruption of this balance postmenopause may further impair vascular healing.^28^^,^^29^

Pharmacologic variability may also be a potential contributor to differences in results. Although current guidelines do not differentiate antiplatelet therapy based on sex, data suggest that men and women may respond differently to commonly used antiplatelet agents. Studies evaluating aspirin have shown conflicting results regarding residual platelet activity, with some reporting higher platelet reactivity in women,^30^ whereas others have shown the opposite or no significant difference,^31^^,^^32^ indicating potential variability across study populations and assay methodologies. An RCT showed that low-dose aspirin reduced the risk of stroke but did not affect MI or the risk of mortality.^33^ In contrast, evidence for sex differences in response to clopidogrel has been more consistent. In the ADAPT-DES study, women were more likely than men to exhibit high residual platelet reactivity while on clopidogrel therapy (51.7% vs 39.6%).^34^ A meta-analysis of 5 RCT showed significant benefit of clopidogrel in reducing cardiovascular death, MI, or stroke in both sexes, but also highlighted reduced benefit in women compared with men (OR 0.93 in women vs 0.84 in men).^35^ These differences in platelet biology and pharmacodynamics may contribute to the elevated risk of complications observed in women following IVBT.

Another contributing factor may be the underlying pattern of ISR. Fibrocellular ISR, characterized by stable neointimal hyperplasia, is more common in women, whereas neoatherosclerotic ISR, involving lipid-laden plaque and greater vulnerability, is more frequently seen in men.^32^^,^^33^ Although fibrocellular ISR is histologically more stable, it can still lead to recurrent revascularization, particularly in women with altered postmenopausal inflammatory responses. In addition to biological mechanisms, social determinants may also contribute to worse outcomes. Women often present later than men with ischemic heart disease due to symptom misattribution and limited awareness of cardiovascular risk.^20^^,^^36^ Provider bias and the labeling of symptoms as “atypical” contribute to underuse of diagnostic testing and guideline-based therapy, even in women with typical presentations.^37^, ^38^, ^39^, ^40^ Together, these pharmacologic, pathophysiological, hormonal, and social factors may explain the increased risk of adverse outcomes observed in women following IVBT, despite the absence of sex-based differences in procedural parameters.

This discrepancy shows that technical success alone may not be sufficient to ensure optimal long-term outcomes in women. The findings suggest that further investigation is needed in women undergoing IVBT. Closer longitudinal follow-up, enhanced adherence to guideline-directed medical therapy, and proactive risk factor modification are critical. Given the multiplicity of secondary end points, these observations warrant confirmation in sex-stratified prospective studies with prespecified primary outcomes and multiplicity control. Furthermore, incorporating sex-based risk stratification into routine clinical decision-making may help identify women at elevated risk early in the treatment course.

This study has several limitations. First, its retrospective and observational design introduces potential for selection bias and residual confounding, despite multivariable adjustment. Second, detailed angiographic data, such as the number of stent layers (single vs multiple), were not available, which limits the ability to assess complexities that may have influenced outcomes.^7^^,^^41^ Third, factors such as medication adherence, menopausal status, and hormonal therapy use, which are potentially relevant to sex-based outcome differences, have not been captured. Fourth, as this was a single-center study with a nonglobal population, the patterns of disease presentations may vary across populations, and it is difficult to determine whether women present later in the course of the disease as compared to men. Additionally, restenosis timing was unavailable for a proportion of patients due to out-of-network index procedures and incomplete archival records; therefore, time to restenosis for target lesion failure could not be explored as a predictor for TLR.

Despite these limitations, the study has the following strengths. It included a well-characterized cohort undergoing IVBT for ISR and a comprehensive evaluation of both procedural and long-term outcomes. Most importantly, it provides novel insights into sex-based differences in this specific clinical context, an area with limited prior data. Future research should prioritize sex-stratified studies with adequate female representation to validate the observed differences in outcomes following IVBT. There is also a need to develop sex-specific risk prediction models to guide postprocedural management and follow-up intensity.

## Conclusion

In this retrospective analysis of patients undergoing IVBT for ISR, female sex was independently associated with higher rates of TLR and MACE, despite comparable rates of procedural success between sexes. Multivariable Cox regression, adjusting for clinical and procedural covariates, confirmed this association. No statistically significant sex-based differences were observed for all-cause mortality, MI, or HFH. These findings show the importance of sex-stratified analyses in interventional studies and support the need for prospective research to better characterize sex-specific risk factors and optimize long-term outcomes following brachytherapy.
